# Tunable Drug Release from 3D-Printed Bilayer Tablets: Combining Hot-Melt Extrusion and Fused Deposition Modeling

**DOI:** 10.3390/polym18020210

**Published:** 2026-01-13

**Authors:** Sangyeob Lee, Eon Soo Song, Eungyeop Lee, Gabin Kwon, Dong Wuk Kim

**Affiliations:** 1 BK21 FOUR Community-Based Intelligent Novel Drug Discovery Education Unit, Vessel-Organ Interaction Research Center (VOICE, MRC), College of Pharmacy, Research Institute of Pharmaceutical Sciences, Kyungpook National University, Daegu 41566, Republic of Korea; sol9438@naver.com (S.L.); djstn0424@naver.com (E.S.S.); sa03103@naver.com (E.L.); 2 UCL School of Pharmacy, University College London, 29–39 Brunswick Square, London WC1N 1AX, UK; gabin.kwon.22@ucl.ac.uk

**Keywords:** theophylline, hot-melt extrusion, fused deposition modeling 3D printing, bilayer tablet, controlled drug release, dissolution profile

## Abstract

This study presents a practical and tunable 3D printing-based approach for manufacturing oral controlled-release bilayer tablets by modulating drug release solely through layer ratio control within a single dosage form. Theophylline-loaded filaments were prepared via hot-melt extrusion (HME) using Kollicoat^®^ IR or hydroxypropyl cellulose as polymer matrices. The mechanical properties of the manufactured filaments were evaluated and compared with commercial filaments to confirm their suitability for fused deposition modeling (FDM) printing. Physicochemical characterization using scanning electron microscopy, differential scanning calorimetry, X-ray diffraction, and Fourier transform infrared spectroscopy indicated partial crystallinity and molecular dispersion of the drug within the polymer matrices. Using a dual-nozzle FDM 3D printer, five bilayer tablets composed of two drug-loaded filaments at different layer ratios were successfully fabricated without altering formulation composition or processing conditions. Drug release studies revealed distinct dissolution behaviors that were strongly dependent on the bilayer composition. Overall, this study demonstrates that controlled drug release can be effectively achieved through geometric modulation of bilayer structures using a combined HME–FDM 3D printing approach, providing a practical platform for personalized oral drug delivery without increasing formulation complexity.

## 1. Introduction

Hot-melt extrusion (HME) is a thermal solventless technology that homogenizes a drug-excipient mixture through multiple operations such as feeding, heating, mixing, and shaping in one continuous process [1]. During the extrusion process, thermal energy is typically provided through the transfer of heat from the barrel and the friction generated between the material and the kneading components, as well as the inner surface of the extruder barrel. Some thermally labile materials may be limited by the effects of thermal, mechanical, and oxidative degradation, but based on prior research, we have demonstrated that they can be successfully fabricated with HME [2,3]. Post-processing steps such as grinding, pelletizing, and tableting are required to obtain the final dosage form. However, this problem can be solved by combining with fused deposition modeling (FDM) 3D printing [1].

In recent years, research interest in 3D printing technology in pharmaceutics has increased following FDA approval for Spiritam [4]. Types of 3D printing include Fused Deposition Modeling [5], Selective Laser Sintering [6], and Inkjet Printing [7,8]. FDM is a 3DP technology that is relatively inexpensive and easily accessible to a broad spectrum of consumers [9]. It also has the advantage of being able to produce formulations with complex geometric structures through software manipulation [10,11]. In this method, the filament from HME goes through a nozzle heated to a high temperature, turning it into a semi-liquid form. It is then laid down and solidifies upon the build platform [5].

Compared to traditional batch manufacturing processes, personalized medicine can be performed through integrating HME and FDM 3D printing, allowing products to be tailored to each patient [12,13]. Therefore, this combination can be seen as a revolutionary change in pharmaceutical manufacturing [14].

The primary goal of controlled-release drugs is to release the drug at the desired rate and for the desired amount of time [15]. Drug release control should consider the in vivo environment and drug release control substances and devices. These controlled-release modulating factors enable site-specific drug delivery [16]. Depending on the drug, controlled-release formulations vary in release patterns, but typical examples include zero-order [17], delayed [18], pulsatile [19], and combinational release [20]. Recognizing these requirements, orally controlled-release systems have increasingly been developed using FDM 3D printing technology, which enables flexible design and precise modulation of drug release profiles.

In this study, theophylline (THEO) was selected as a model drug because of its narrow therapeutic window, rapid gastrointestinal absorption, and the resulting difficulty in maintaining stable plasma concentrations, making it a representative candidate for evaluating controlled-release strategies [21,22]. Kollicoat IR was chosen as a rapidly disintegrating, water-soluble polymer to promote immediate drug release [23], whereas low-substituted hydroxypropyl cellulose (L-HPC) was selected as a relatively slowly disintegrating polymer to retard drug release [24]. The distinct disintegration behaviors of these polymers enable the design of bilayer tablets with differentiated release functions within a single dosage form. Both polymers exhibit suitable thermoplastic properties for HME and subsequent FDM 3D printing, which have been widely reported as effective manufacturing approaches for controlled-release oral dosage forms [25]. Unlike conventional bilayer tablets that typically contain different active pharmaceutical ingredients, the present study employs a single API distributed across two layers with different release characteristics. By combining HME and FDM technologies, this approach overcomes common manufacturing issues of bilayer tablets, such as poor interlayer adhesion, and allows controlled-release delivery systems to be fabricated by tailoring the proportion of each layer [7].

## 2. Materials and Methods

### 2.1. Materials

THEO was purchased from Tokyo Chemical Industry (Tokyo, Japan). THEO is crystalline and considered a class I compound in the BCS and has a melting point of 275 °C. L-type HPC, a water-insoluble but swellable polymer, was donated by Hanmi Pharmaceutical (Hwaseong, Republic of Korea). Kollicoat IR was donated by the BASF (Ludwigshafen, Germany). PEG 1500 was supplied by Daejeong Chemical (Siheung, Republic of Korea) and was used as a plasticizer. Commercial PVA and PLA filaments (diameter: 1.75 mm) were purchased from Shenzhen Esun Industrial Co. (Shenzhen, China) and used as reference materials for printability evaluation. All other reagents were of high-performance liquid chromatography (HPLC) or analytical grade and were used as received without further purification.

### 2.2. Preparation of THEO-Loaded Filaments

THEO, HPC, Kollicoat IR, and PEG 1500 were mixed in two compositions (Table 1) using a conventional blender (Tablet Crusher, AGM-05, Ireatech, Daejeon, Republic of Korea). Each batch was blended for 30 s to achieve homogeneous mixing. The mixture was then extruded using a twin-screw extruder (HAAKE™ MiniCTW Micro-Conical Twin-screw Compounder, Thermo Scientific™, Waltham, MA, USA; NFEC-2021-12-275522) at 155–185 °C and a screw speed of 35 rpm through a 1.8 mm nozzle at the Medibio Core Facility of Kyungpook National University. The resulting filament was stored in a vacuum desiccator before printing. THEO loading in the filaments was determined using HPLC analysis.

### 2.3. Texture Analysis Method

**Figure 1:**
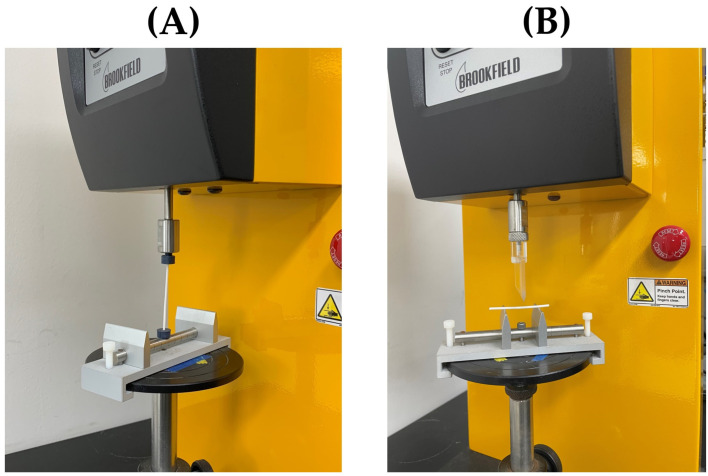
Texture analysis setup for filament mechanical evaluation: (**A**) axial compression (bending simulation) test and (**B**) three-point bending test.

Filament mechanical behavior relevant to FDM printability was evaluated using a texture analyzer (CT3 Texture Analyzer, AMETEK Brookfield, Middleboro, MA, USA) by conducting both axial compression testing and a three-point bending test. For the axial compression test, the MakerBot Replicator 2× has a roller travel speed of approximately 3.20 mm/s and a target displacement of 15 mm, applying axial compression in a displacement-controlled mode. A 50 mm filament was fixed in the custom fixture, and a bending simulation was conducted (Figure 1A). The three-point bending test was used to assess filament flexibility, brittleness, and stiffness, which are key determinants of filament feedability during FDM printing, as previously reported in the literature [26]. For the three-point bending test, filaments with a length of 45 mm were placed on a sample holder with a support span of 25 mm. The test was conducted at a crosshead speed of 0.5 mm/s with a trigger force of 5.0 g and an endpoint displacement of 10 mm (Figure 1B). Commercial PLA and PVA filaments, together with the HME-produced HPC- and Kollicoat IR-based filaments, were evaluated under identical conditions to enable direct comparison of deformation behavior. This approach follows established methodologies for pharmaceutical filament printability assessment, where feedability is interpreted based on bending response rather than absolute force values alone [26]. All texture analysis measurements were performed in triplicate for each filament type.

### 2.4. Fabrication of FDM 3DP Tablets

Dosage forms (F1–F5) were fabricated with the drug-loaded filament using a standard FDM 3D printer (MakerBot Replicator 2× Desktop, MakerBot Inc., Brooklyn, NY, USA) equipped with a dual-nozzle system. Two different drug-loaded filaments were loaded into separate nozzles, and bilayer tablets were produced by sequential deposition, in which one nozzle printed the first layer, followed by the second nozzle printing the subsequent layer. For each formulation, 20 tablets were fabricated for subsequent characterization and dissolution studies. The template used to print the dosage form was designed using Tinkercad 2018 (Autodesk Inc., San Rafael, CA, USA) and exported as a stereolithography (.stl) file into MakerWare v. 3.10.0 (MakerBot Inc., USA). The selected geometry of the dosage forms was a flat-faced cylindrical tablet with dimensions of X = 10 mm, Y = 10 mm, and Z = 5 mm. The infill proportion was 100% to produce solid dosage forms of high density. Other printer settings were as follows: standard resolution with the raft option deactivated and an extrusion temperature of 205–215 °C, platform temperature of 130 °C, speed while extruding of 90 mm/s, speed while traveling of 150 mm/s, two shells, and layer height of 0.20 mm. The diameter and height of the printed tablets were measured using digital Vernier calipers, while the tablet weight was determined using an analytical balance. For each formulation, measurements were performed on multiple samples (*n* = 6), and the results are reported as mean ± standard deviation.

### 2.5. Physicochemical Characterization

#### 2.5.1. Analytical Methods

A Dionex UltiMate 3000 HPLC system (Thermo Fisher Scientific, Germering, Germany; NFEC-2025-09-308581 at the Medibio Core Facility of Kyungpook National University) equipped with an LPG-3400SD pump, WPS-3000TSL autosampler, TCC-3000SD column compartment, and VWD-3100 detector (Thermo Fisher Scientific, Germering, Germany) was used. In addition, a Capcell Pak C18 column (Shiseido, Tokyo, Japan; 250 × 4.6 mm, 5 µm) was used, with the detection wavelength set at 270 nm. Acetonitrile and 50 mM sodium acetate in water at a ratio of 15:85 (*v*/*v*) were used as a mobile phase. The mobile phase flow rate was maintained at 1.0 mL/min, and an injection volume of 10 μL was used. The HPLC data were analyzed using Chromeleon 7 software.

#### 2.5.2. Determination of Drug Loading of the Filaments and 3DP Tablets

To determine the THEO content, the filaments were cut into small pieces and pulverized to obtain a sufficient amount of powder, while each 3DP tablet was pulverized individually due to its higher unit weight (approximately 450 mg). From the resulting powder, approximately 0.1 g was accurately weighed for analysis. To ensure complete drug release, the powder was placed in a 100 mL volumetric flask containing methanol (HPC Filament, tablets) and distilled water (Kollicoat IR Filament, tablets) under magnetic agitation until complete dissolution. The solution was then diluted (10-fold), and the concentration of THEO was measured using HPLC-UV 270 nm (*n* = 3).

#### 2.5.3. Scanning Electron Microscopy (SEM) Analysis

The morphology and surface of the printlets were evaluated individually using a scanning electron microscope (SU-8220; Hitachi, Tokyo, Japan) operated at an acceleration voltage of 5.0 kV. Before analysis, the sample was adsorbed on double-sided adhesive tape and attached to a brass holder. The samples were coated with platinum in vacuo for 4 min using the EmiTeck sputter coater K575 K (Quorum Technologies Ltd., West Sussex, UK).

#### 2.5.4. Differential Scanning Calorimetry (DSC) Analysis

The thermal properties were analyzed using a differential scanning calorimeter (TA Instruments DSC Q10; TA Instruments, New Castle, DE, USA). Approximately 3.8 mg of the sample was weighed, sealed, and placed in an aluminum pan. The sample was heated from 35 °C to 300 °C with a constant temperature change of 10 °C/min.

#### 2.5.5. Powder X-Ray Diffraction (PXRD) Analysis

Powder X-ray diffraction of all powder samples (plain drug, polymers, and their respective powder mixtures) was carried out using a D/Max-2500 X-ray diffractometer (Rigaku, Oxford, UK) equipped with a Cu Kα radiation source at 40 KV voltage and 200 mA current. Diffraction patterns were obtained in the 2θ range of 5–50° using 0.05 step sizes.

#### 2.5.6. Fourier Transform Infrared (FTIR) Analysis

A Fourier transform infrared spectrophotometer (Cary 630 FTIR; Agilent Technologies, Santa Clara, CA, USA) was used. The wavelength was scanned from 500 to 4000 cm^−1^ with a resolution of 2 cm^−1^. THEO, HPC, Kollicoat IR, filament, printed tablet, and physical mixtures (PMs) were also evaluated.

### 2.6. In Vitro Dissolution

The in vitro drug release behavior of THEO HPC tablets and Kollicoat IR tablets was designed to simulate gastrointestinal pH transition conditions and was first evaluated for 2 h in 750 mL of 0.1 N HCl (pH 1.2), corresponding to the gastric environment. After 2 h, the dissolution medium was shifted to intestinal conditions by the addition of 250 mL of 0.2 M trisodium phosphate, and the pH was adjusted to 6.8 using 2 N sodium hydroxide to reflect the physiological pH of the small intestine, where the majority of drug absorption occurs. The 2 h transition time was selected based on the typical gastric residence time for solid oral dosage forms under fasted conditions. In addition, an in vitro drug release study was performed on all 3DP tablets by the USP dissolution apparatus 2, paddle method. The dissolution tester (DT 620; ERWEKA, Heusenstamm, Germany) maintained a stirring speed of 50 rpm and a constant temperature of 36.5 ± 0.5 °C. At predetermined time intervals (5, 10, 15, 30, 60, 90, and 120 min, followed by hourly sampling after pH transition), an aliquot (approximately 1.5 mL) was sampled and filtered through a 0.45 µm PTFE membrane syringe filter. Afterward, the quantification of the drug in the samples was determined by the HPLC, as described above. The dissolution test was performed in triplicate (*n* = 3).

## 3. Results and Discussion

### 3.1. Preparation of Drug-Loaded THEO Filaments

Material selection is important when developing filaments using FDM 3DP. The delivery of the API using HME required appropriate polymeric carriers. However, most pharmaceutical-grade polymers commonly used for the oral dosage forms are not extruded into the desired filament form for printing with HME. Therefore, the HME process places various requirements on polymers based on their mechanical properties.

HPC, chemically known as cellulose 2-hydroxypropyl ether, is commonly used as a thickener, tablet binder, film coating, and extended-release matrix in oral dosage forms. The temperature of the low-glass transition HPC makes it flexible and easy to extrude through an HME [14]. Long rod-shaped filaments were formed by mixing the THEO drug, cellulosic polymer, and plasticizer PEG 1500 using twin-screw HME. The temperature was maintained at 155 °C, and the HPC filament with THEO had a solid white appearance. Filaments with consistent diameters and reasonable flexibility were manufactured, ranging from 1.66 ± 0.01 mm.

Second, Kollicoat IR, also known as macrogol-poly (vinyl alcohol) graft-copolymer, was used as a pore-forming, emulsifying, binding, film-forming, and immediate-release coating material. Because this product is non-ionic, its solubility does not change as the pH increases or decreases along the gastrointestinal tract [27]. The Kollicoat IR and THEO are polar. The closer the polarity values between the two compounds, the higher the affinity [28,29]. Therefore, THEO was well-suited for extruding HME by integrating it with Kollicoat IR, and it was easy to extrude. The temperature was maintained at 185 °C, and the Kollicoat IR filament with THEO had a solid yellow appearance. The diameters ranged from 1.70 (±0.009) mm, resulting in very consistent diameters and flexibility.

### 3.2. Texture Analysis Study

The mechanical behavior of the HME-produced filaments was interpreted in terms of FDM feedability by jointly considering the axial compression and three-point bending results summarized in Table 2. The axial compression resistance and peak load values of both HPC- and Kollicoat IR-based filaments were positioned between those of commercial PVA and PLA filaments, indicating intermediate stiffness relative to commonly used FDM reference materials and sufficient robustness to withstand feeding pressure without excessive rigidity.

Notably, the breaking distance obtained from the three-point bending test, which is closely associated with filament flexibility and resistance to brittle fracture, was comparable to that of commercial PLA filaments. This suggests that the prepared filaments can tolerate bending deformation during feeding without premature breakage, supporting stable and continuous extrusion. Overall, these results demonstrate a balanced combination of stiffness and flexibility conducive to reliable feedability, consistent with previously reported printability assessment frameworks that emphasize bending behavior rather than absolute force magnitude [26].

### 3.3. 3DP of THEO Dosage Forms

Cylindrical tablets designed using CAD programs were successfully printed using 3DP. As a characteristic of FDM printers, they are easy to handle, so their convenience is very high, and the result is also very satisfactory. As previously reported, 3DP tablets have very high strength, thus, have a shape similar to that of plastic-coated tablets [30,31]. Therefore, it was difficult to quantify the hardness using a hardness tester. The friability of all formulations was zero, and it was very difficult to separate them.

In the process of 3DP, the temperature was higher than that of the actual HME. This is because of the differences in the heating rates. When manufacturing filaments using HME, the processing temperature can be maintained for more than 20 min. However, when using a 3D printer, the temperature of the printer nozzle is much higher than that of the HME because the hard filament must be made into a semi-solid state at an instantaneous high temperature. The filament passes through the hot nozzle of the printer at a high speed. After these filaments are formed into a semi-solid state, rapid solidification occurs owing to the relative room temperature.

**Figure 2:**
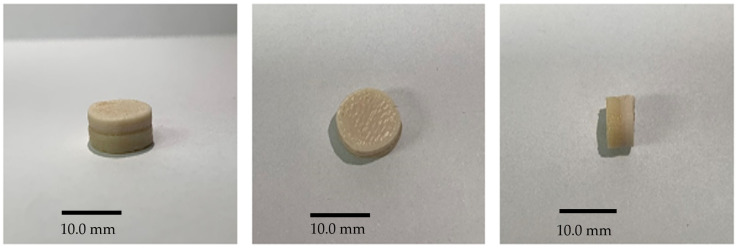
Three-dimensional-printed bilayer tablet formed of 50% hydroxypropyl cellulose and 50% Kollicoat IR.

As shown in Figure 2, it was confirmed that the bilayer tablet of HPC and Kolicoat IR has been formed without difficulty even when the nozzle temperature of the 3D printer increased. The set nozzle temperatures of the 3D printer were 215 °C and 205 °C, which was possible because the melting point of THEO was as high as 275 °C. However, if the nozzle temperature was lower than this set value, the solid-semi-solid process would become difficult; therefore, the tablet would not accumulate properly, and manufacturing would stop. Tablets with consistent diameters and heights were produced (Table 3).

### 3.4. Physicochemical State Characterization

#### 3.4.1. Determination of Drug Loading

The chemical integrity of the drug in the 3D-printed tablets and filaments was analyzed using HPLC. The drug loading for HPC filaments was 21.84 ± 0.43 μg/mL (theoretical loading: 20 μg/mL), and the drug loading for Kollicoat IR filaments was 21.52 ± 0.50 μg/mL (theoretical loading: 20 μg/mL). The drug loading for the printed HPC tablets (F5) was 21.30 ± 0.04 μg/mL (theoretical loading: 20 μg/mL), corresponding to an actual drug content of 94.02 mg per tablet compared with a theoretical value of 88.28 mg per tablet. Similarly, the Kollicoat IR tablets (F1) showed a drug loading of 22.16 ± 2.74 μg/mL (theoretical loading: 20 μg/mL), corresponding to an actual content of 102.93 mg per tablet versus a theoretical value of 92.89 mg per tablet, indicating that no drug loss occurred during processing.

#### 3.4.2. Scanning Electron Microscopy (SEM) Results

**Figure 3:**
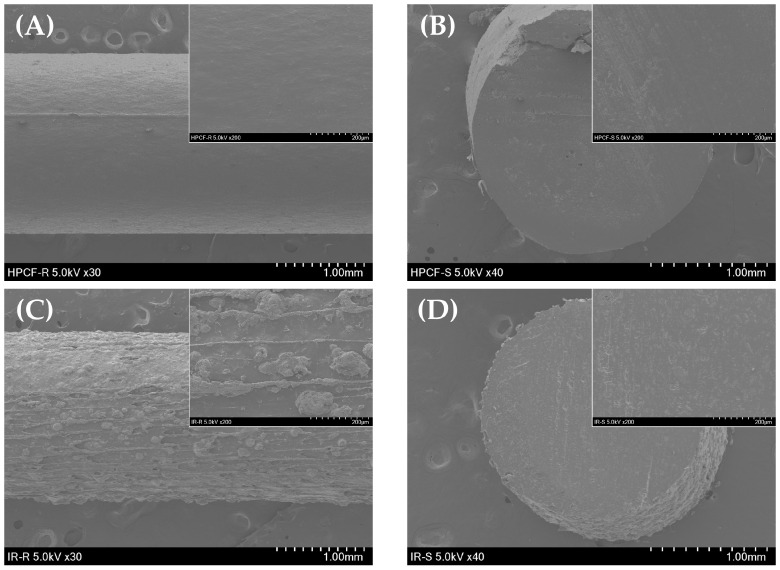
SEM images of (**A**) THEO-loaded HPC filament exterior (×30) and (**B**) cross-sectional shape (×40) and (**C**) THEO-loaded Kollicoat IR filament exterior appearance (×30) and (**D**) cross-sectional shape (×40). Higher-magnification SEM images (×200) are shown in the upper right corner of each panel.

SEM was performed to closely observe the structures of the extruded HPC, Kollicoat IR filament, and 3D-printed tablet. As shown in Figure 3, the HPC filament extruding through HME had a rod shape and smooth surface, and no special pores were observed. Kollicoat IR filaments had a branch-like shape, a very rough surface.

**Figure 4:**
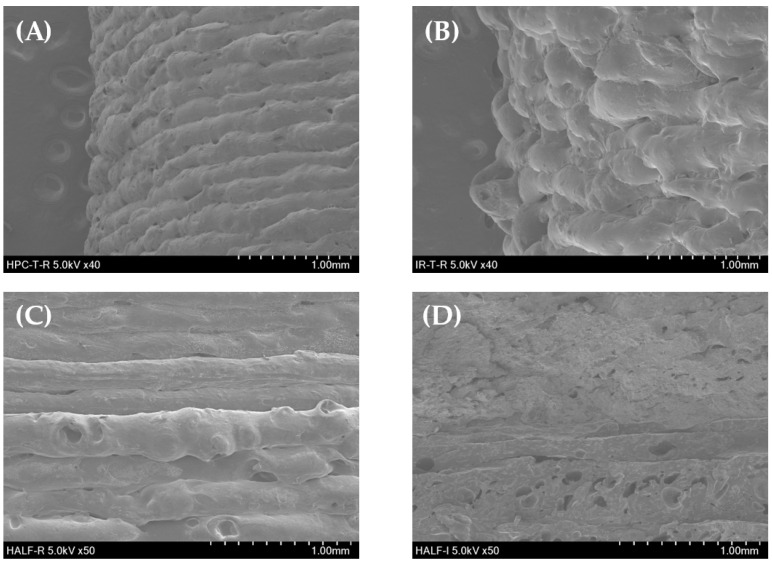
SEM images of (**A**) THEO-loaded HPC 3D-printed tablet exterior (×40), (**B**) THEO-loaded Kollicoat IR 3D-printed tablet exterior (×40), and (**C**) THEO-loaded bilayer tablet exterior (×50) and (**D**) cross-sectional shape (×50).

Figure 4 shows the surface and cross-section of the 3D-printed tablet and the bilayer tablet, and it was found that the stacking pattern of HPC and Kollicoat IR tablets and the size of the porous cross-section were slightly different. As for the layering aspect, it was observed that HPC was deposited more precisely than Kollicoat IR. In the 3DP process, the smooth surface of the HPC filament is very suitable for applying constant temperature and pressure, and consistent and dense lamination may be possible. On the other hand, the Kollicoat IR filament has a very rough surface; therefore, it is expected that the compactness will be poor because it is difficult to achieve consistent lamination.

Both HPC and Kollicoat IR filament layers were confirmed to have small pores dispersed in a dense form. The impact of PEG 1500 is obvious. PEG is a hydrophilic polymer that acts as a pore former [32,33]. Thus, porosity was observed in several places in the HPC and Kollicoat IR tablets. It has been demonstrated that the presence of hydrophilic polymers in the matrix system induces pore formation, which increases the moisture content of the particles or improves the dissolution and diffusion of drugs. As shown in Figure 4D, transparent pores are formed in the Kollicoat IR layer [28]. While HPC is generally water-soluble, a high degree of hydroxypropyl substitution imparts thermoplastic characteristics and reduces its effective hydrophilicity compared with less-substituted cellulose derivatives. In contrast, L-HPC, owing to its limited substitution, is insoluble in water and primarily exhibits swelling- and disintegration-promoting behavior rather than film-forming properties. This extensive degree of hydroxypropylation imparts to HPC somewhat more hydrophobic properties [34]. Thus, the pore size of HPC was smaller than that of the Kollicoat IR layer.

#### 3.4.3. Differential Scanning Calorimetry (DSC) Results

**Figure 5:**
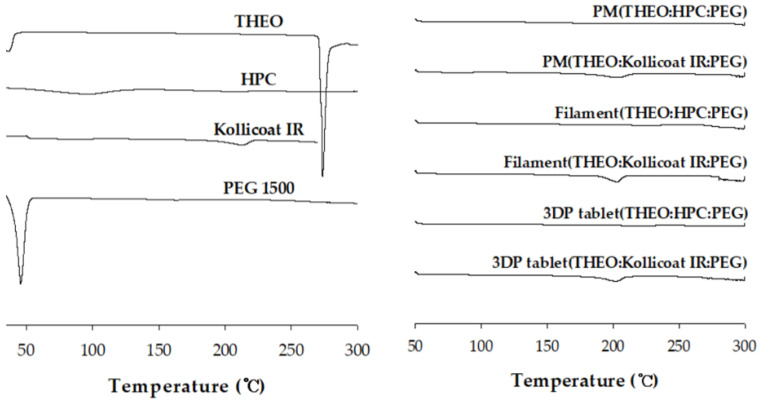
DSC curves for the samples. THEO, theophylline; HPC, hydroxypropyl cellulose; PEG, polyethylene glycol; PM, physical mixture; 3DP, three-dimensional printing.

Thermal behaviors of pure substances, HME filaments, and 3D printed tablets are presented in Figure 5. The DSC thermogram showed a characteristic melting endotherm for THEO (275 °C), which is in good agreement with the literature [35]. However, owing to the introduction of HPC and Kollicoat IR, specific endothermic peaks were not observed for the PM, HPC, and Kollicoat filament, and HPC and Kollicoat 3D-printed tablets. The result of DSC analysis is that the dispersion of THEO within the HPC and Kollicoat IR polymer matrix may result in reduced crystalline form. Similar suppression of the theophylline melting endotherm after HME–FDM processing has been widely reported and is commonly attributed to molecular dispersion or partial amorphization of the drug within polymer matrices [36]. Therefore, PXRD proceeded further for thermal analysis.

#### 3.4.4. Powder X-Ray Diffraction (PXRD) Results

**Figure 6:**
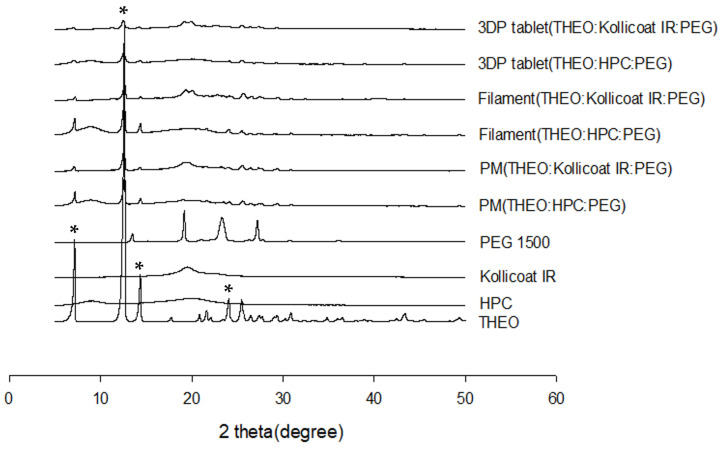
PXRD curves for samples. 3DP, three-dimensional printing; HPC, hydroxypropyl cellulose; PEG, polyethylene glycol; PM, physical mixture; THEO, theophylline. Asterisks (*) indicate the characteristic diffraction peaks of crystalline theophylline.

Because the processing temperatures of HME and 3DP are very high, heat-labile drugs or used excipients may be decomposed. PXRD was performed to confirm the physical state of the drug. Figure 6 shows that THEO exhibits prominent peaks at approximately θ = 7, 12, 14 and 24, which are marked with asterisks (*), indicating its high crystallinity. Unexpectedly, the results of PM, filament, and 3DP tablet from PXRD were different from those from DSC thermograms. PM, filament, and 3DP tablets displayed a number of peaks, all of which had a declining intensity. This may be because the low-resolution DSC thermograms did not show crystallinity below 2% [14,37].

These observations suggest that the THEO crystals have been converted to a partially crystalline state with molecular dispersion of the drug into the polymer matrix.

#### 3.4.5. Fourier Transform Infrared (FTIR) Results

**Figure 7:**
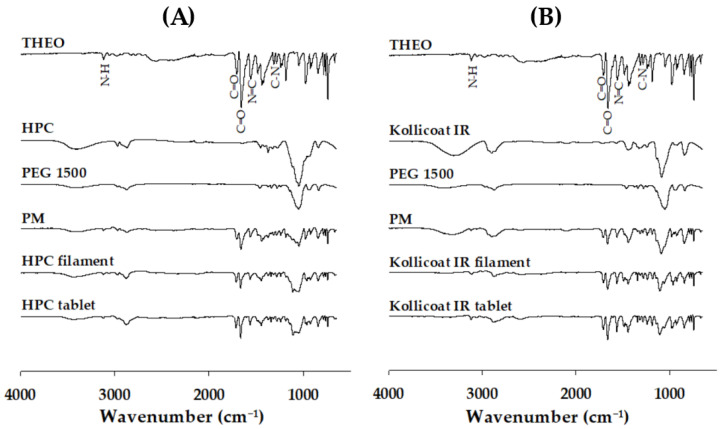
FTIR spectra of the THEO, polymers, PEG 1500, physical mixtures, filaments, and 3DP tablets related to (**A**) HPC or (**B**) Kollicoat IR. THEO, theophylline; HPC, hydroxypropyl cellulose; PEG, polyethylene glycol; PM, physical mixture.

The FTIR profiles were also utilized to explore the presence of a THEO within filaments and printed tablets. The spectrum of crystalline THEO showed characteristic peaks at 1670~1660 cm^−1^ and 1717~1716 cm^−1^ (C=O stretching), 3500~3100 cm^−1^ and 1560–1640 cm^−1^ (N-H stretching), 1566 cm^−1^ (N=C stretching) and 1320~1300 cm^−1^ (C–N stretching of the xanthine ring). The FTIR results showed that there was no interaction between THEO, HPC, and Kollicoat IR in the filament and 3D-printed tablet (Figure 7). An important functional group of the drug remains undetected when the interaction occurs. Therefore, the main IR peak of THEO was observed in the 3DP spectrum, indicating that the molecular structure of THEO was not completely damaged. This result indicates that there is no perceived interaction between the drug and the excipient.

### 3.5. In Vitro Dissolution Study

**Figure 8:**
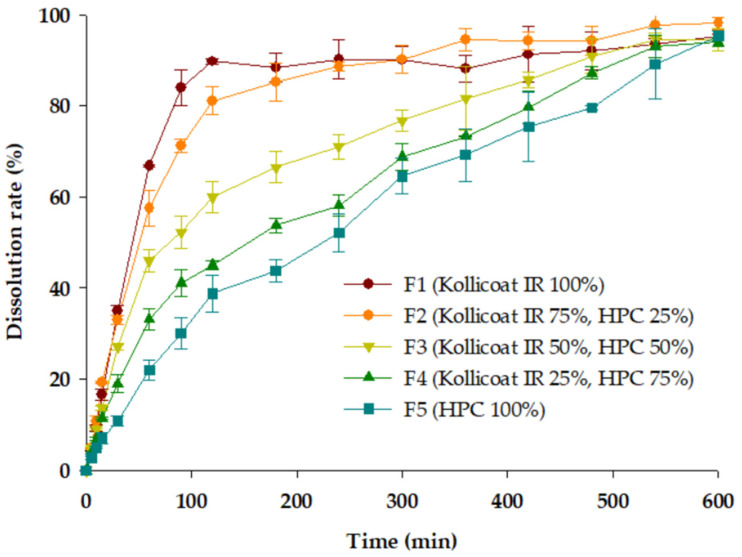
Drug release profiles of the three-dimensional-printed tablets. Each value represents the mean ± standard deviation (*n* = 3). HPC, hydroxypropyl cellulose.

**Figure 9:**
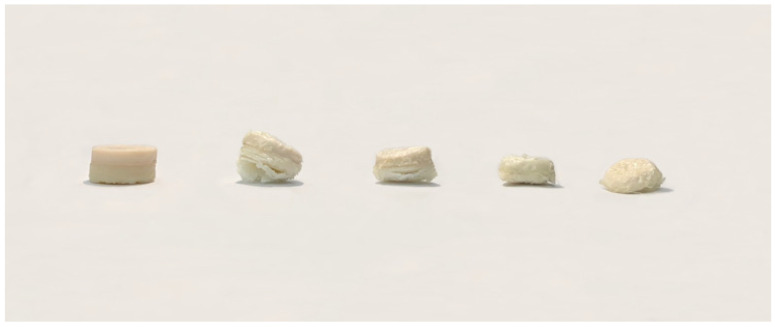
Changes in the dissolution time (0, 30, 60, 90, and 120 min) of Kollicoat IR 50% and hydroxypropyl cellulose 50% three-dimensional-printed bilayer tablet.

THEO is classified as a class l molecule in the BCS [38]. Therefore, it has high solubility and bioavailability. To distribute the highly soluble THEO into various dissolution profiles, the ratio of excipient HPC, the Kollicoat IR matrix, and the bilayer tablet was changed. The dissolution profiles of the 3D-printed formulations prepared by varying the ratio of the first and second layers were compared (Figure 8), and a clear difference was observed between them. F1 exhibited almost 100% dissolution within 120 min. In contrast, F5 showed a sustained release profile of 600 min up to 100% dissolution. Consequently, the dissolution profile of the Kollicoat IR matrix was faster than that of the HPC matrix. F2, F3, and F4 showed a faster dissolution profile as the Kollicoat IR ratio increased. However, when the HPC ratio was high, a slightly slower dissolution profile was observed. Figure 9 shows the changes in the properties of the (0, 30, 60, 90, and 120 min) bilayer tablet of F3 with dissolution time. It was observed that Kollicoat IR with large pores was rapidly dissolved (first-layer Kollicoat IR).

The dense F5 HPC tablet exhibited a significantly prolonged drug release rate, whereas the F1 Kollicoat IR tablet exhibited faster drug release. Kollicoat IR is a polymer that rapidly disintegrates through the rapid absorption of water (dissolution medium) and swelling. As confirmed through SEM, the surface of the Kollicoat IR tablet had a large number of pores, so penetration of the dissolution medium was easy. On the other hand, compared to other water-soluble polymers, HPC is plastic and hydrophobic, thus, has lower porosity, and decreases the inflow of the dissolution medium, leading to a decrease in solubility. Differences between the two matrix polymers were evident owing to the characteristics of these excipients. The remaining F2, F3, and F4 are simple but can be controlled by changing the ratio between the layers.

Although all five THEO 3D-printed bilayer tablets exceeded the tablet hardness limit, the in vitro dissolution profile showed an adequate dissolution pattern, with 80% drug release within 10 h.

Overall, the dissolution behavior of the bilayer tablets can be mechanistically interpreted based on distinct release-controlling mechanisms associated with each polymeric layer. Drug release from the Kollicoat IR layer was dominated by rapid disintegration and diffusion-controlled processes, whereas the HPC layer followed a matrix-controlled diffusion mechanism. In the bilayer systems, the relative contribution of each layer dictated the overall release behavior, demonstrating that controlled adjustment of layer ratios enables predictable tuning of drug release mechanisms from immediate-release–dominated to sustained-release–dominated profiles.

## 4. Conclusions

This innovative study successfully produced an orally controlled-release bilayer tablet, providing an alternative method for fabricating a controlled-release delivery system by altering the proportions of the layers. Physical characterization showed that the crystalline drug has been converted to a partially crystalline state with molecular dispersion of the drug into the polymer matrix. Different dissolution profiles allow for the local or systemic delivery of a given drug to specific regions of the stomach and intestine. Therefore, the main finding of this study is that the combination of novel HME- and FDM-based 3DP technology has good controlled-release rates, and it could be beneficial for the development of personalized medicines.

## Figures and Tables

**Table 1:** Composition of drug-loaded filaments and extrusion temperature.

Formulation	THEO (%)	HPC (%)	Kollicoat IR (%)	PEG 1500 (%)	Extrusion Temperature (°C)
Filament K	20	-	75	5	185
Filament H	20	75	-	5	155

HPC, hydroxypropyl cellulose; PEG, polyethylene glycol; THEO, theophylline.

**Table 2:** Mechanical properties of commercial and HME-produced filaments evaluated by axial compression and three-point bending tests (mean ± SD, *n* = 3).

Filament	Axial Compression Test	Three-Point Bending Test	
	Force (g)	Breaking Distance (mm)	Peak Load (g)
PVA (commercial)	219.33 ± 69.83	6.54 ± 0.86	133.42 ± 15.13
Kollicoat IR	354.67 ± 88.30	2.52 ± 0.16	191.38 ± 6.59
HPC	415.17 ± 65.29	2.88 ± 0.26	258.19 ± 5.90
PLA (commercial)	536.83 ± 42.32	3.16 ± 0.01	717.41 ± 12.52

**Table 3:** Physical properties of the three-dimensional-printed tablets (mean ± SD, *n* = 6).

	Layer Ratio (%)	Radius (mm)	Height (mm)	Weight (mg)
F1	Kollicoat 100	10.23 ± 0.23	5.26 ± 0.16	464.47 ± 13.71
F2	HPC 25, Kollicoat IR 75	10.17 ± 0.14	5.19 ± 0.07	470.67 ± 5.97
F3	HPC 50, Kollicoat IR 50	10.37 ± 0.13	5.30 ± 0.10	455.60 ± 3.68
F4	HPC 75, Kollicoat IR 25	10.21 ± 0.07	5.23 ± 0.04	463.74 ± 5.18
F5	HPC 100	10.17 ± 0.11	5.18 ± 0.06	441.42 ± 3.32

## Data Availability

The original contributions presented in the study are included in the article; further inquiries can be directed to the corresponding author.
